# No Treatment versus 24 or 60 Weeks of Antiretroviral Treatment during Primary HIV Infection: The Randomized Primo-SHM Trial

**DOI:** 10.1371/journal.pmed.1001196

**Published:** 2012-03-27

**Authors:** Marlous L. Grijsen, Radjin Steingrover, Ferdinand W. N. M. Wit, Suzanne Jurriaans, Annelies Verbon, Kees Brinkman, Marchina E. van der Ende, Robin Soetekouw, Frank de Wolf, Joep M. A. Lange, Hanneke Schuitemaker, Jan M. Prins, on behalf of the Primo-SHM Study Group

**Affiliations:** 1 Department of Internal Medicine, Division of Infectious Diseases, Center for Infection and Immunity, Academic Medical Center, University of Amsterdam, Amsterdam, The Netherlands; 2 Department of Global Health, Amsterdam Institute for Global Health and Development, Academic Medical Center, University of Amsterdam, Amsterdam, The Netherlands; 3 Department of Medical Microbiology, Academic Medical Center, University of Amsterdam, Amsterdam, The Netherlands; 4 Department of Medical Microbiology, Maastricht University Medical Center, Maastricht, The Netherlands; 5 Department of Internal Medicine, Onze Lieve Vrouwe Gasthuis, Amsterdam, The Netherlands; 6 Department of Internal Medicine, Erasmus Medical Center, Rotterdam, The Netherlands; 7 Department of Internal Medicine, Kennemer Gasthuis, Haarlem, The Netherlands; 8 HIV Monitoring Foundation, Amsterdam, The Netherlands; 9 Department of Experimental Immunology, Academic Medical Center, University of Amsterdam, Amsterdam, The Netherlands; Johns Hopkins Bloomberg School of Public Health, United States of America

## Abstract

**Background:**

The objective of this study was to assess the benefit of temporary combination antiretroviral therapy (cART) during primary HIV infection (PHI).

**Methods and Findings:**

Adult patients with laboratory evidence of PHI were recruited in 13 HIV treatment centers in the Netherlands and randomly assigned to receive no treatment or 24 or 60 wk of cART (allocation in a 1∶1∶1 ratio); if therapy was clinically indicated, participants were randomized over the two treatment arms (allocation in a 1∶1 ratio). Primary end points were (1) viral set point, defined as the plasma viral load 36 wk after randomization in the no treatment arm and 36 wk after treatment interruption in the treatment arms, and (2) the total time that patients were off therapy, defined as the time between randomization and start of cART in the no treatment arm, and the time between treatment interruption and restart of cART in the treatment arms. cART was (re)started in case of confirmed CD4 cell count <350 cells/mm^3^ or symptomatic HIV disease. In total, 173 participants were randomized. The modified intention-to-treat analysis comprised 168 patients: 115 were randomized over the three study arms, and 53 randomized over the two treatment arms. Of the 115 patients randomized over the three study arms, mean viral set point was 4.8 (standard deviation 0.6) log_10_ copies/ml in the no treatment arm, and 4.0 (1.0) and 4.3 (0.9) log_10_ copies/ml in the 24- and 60-wk treatment arms (between groups: *p*<0.001). The median total time off therapy in the no treatment arm was 0.7 (95% CI 0.0–1.8) y compared to 3.0 (1.9–4.2) and 1.8 (0.5–3.0) y in the 24- and 60-wk treatment arms (log rank test, *p*<0.001). In the adjusted Cox analysis, both 24 wk (hazard ratio 0.42 [95% CI 0.25–0.73]) and 60 wk of early treatment (hazard ratio 0.55 [0.32–0.95]) were associated with time to (re)start of cART.

**Conclusions:**

In this trial, temporary cART during PHI was found to transiently lower the viral set point and defer the restart of cART during chronic HIV infection.

**Trial registration:**

Current Controlled Trials ISRCTN59497461

*Please see later in the article for the Editors' Summary*

## Abstract 2

In a three-arm randomized trial conducted among adult patients in HIV treatment centers in The Netherlands, Marlous Grijsen and colleagues examine the effects of temporary combination antiretroviral therapy during primary HIV infection.

## Editors' Summary

**Background:**

Every year, nearly three million people become infected with HIV, the virus that causes AIDS. The first stage of HIV infection—primary HIV infection—lasts a few weeks and often goes undetected, although most individuals develop a short, flu-like illness. During this stage of infection, the immune system begins to make antibodies to HIV. The second stage of HIV infection, which lasts many years, also has no major symptoms but, during this stage, HIV slowly destroys immune system cells, including CD4 cells, a type of lymphocyte. Eventually, the immune system is unable to fight off other infections and patients enter the third phase of HIV infection—symptomatic HIV infection. The final stage—AIDS—is characterized by the occurrence of one or more AIDS-defining conditions, which include severe but unusual infections and several types of cancer. Early in the AIDS epidemic, most HIV-positive people died within ten years of infection. Nowadays, although there is still no cure for HIV infection, HIV has become a chronic disease because of the availability of combination antiretroviral therapy (cART; cocktails of several powerful drugs). This means that many HIV-positive people have a near-normal life span.

**Why Was This Study Done?:**

It is currently recommended that people start cART when their CD4 count falls below 350 CD4 cells per cubic milliliter (cells/mm^3^) of blood, when they develop severe constitutional symptoms such as fever lasting longer than a month, or when they develop an AIDS-defining condition. But could a short course of cART during primary HIV infection be clinically beneficial? Some, but not all, nonrandomized studies have shown that such treatment reduces the viral set point (the stabilized viral load that is reached after the immune system begins to make antibodies to HIV; the viral load is the amount of virus in the blood) and slows the decline of CD4 cell count in patients. In this randomized trial (the Primo-SHM trial), the researchers assess the clinical benefit of temporary cART initiated during primary HIV infection by measuring its effects on the viral set point and on when patients have to restart cART during chronic HIV infection. In a randomized controlled trial, patients are assigned by the play of chance to receive different treatments and then followed to compare the effects of these treatments.

**What Did the Researchers Do and Find?:**

The researchers assigned 168 patients with primary HIV infection to receive no treatment, 24 weeks of cART, or 60 weeks of cART. They measured the viral set point (the viral load in the blood 36 weeks after randomization in the no treatment arm and 36 weeks after cART interruption in the treatment arms) and determined the time off therapy (the time between randomization and the start of cART in the no treatment arm, and the time between treatment interruption and restart of cART in the treatment arms) for each patient. cART was (re)started following two consecutive CD4 counts below 350 cells/mm^3^ or when symptomatic HIV disease developed. The average viral set point was lower in the patients who received early cART than in those who had no treatment. Moreover, on average, the patients in the no treatment arm started cART 0.7 years after randomization whereas those in the 24- and 60-week treatment arms restarted cART after 3.0 and 1.8 years, respectively. There was no statistically significant difference between the 24-week and 60-week treatment arms in time off therapy.

**What Do These Findings Mean?:**

These findings suggest that temporary cART during primary HIV infection can transiently lower the viral set point and can delay the need to restart cART during chromic HIV infection. They also suggest that 24 weeks of cART during primary HIV is as effective as 60 weeks of treatment. These findings need to be confirmed in other settings, and follow-up studies are needed to evaluate the long-term benefits of early temporary cART, but given the short time between cART interruption and treatment restart, the researchers suggest that not interrupting early cART, but instead continuing it for life, should be considered. However, they add, because patients are often physically and emotionally distressed at this stage of HIV infection, adherence to cART during primary HIV infection may be suboptimal, and so patients with primary HIV infection should be advised to start cART only when they feel ready to start treatment.

**Additional Information:**

Please access these web sites via the online version of this summary at http://dx.doi.org/10.1371/journal.pmed.1001196.

- Information is available from the US National Institute of Allergy and infectious diseases on HIV infection and AIDS, including information on the clinical progression of HIV infection
- NAM/aidsmap provides information about HIV/AIDS, including a factsheet on primary infection
- Information is available from Avert, an international AIDS charity on many aspects of HIV/AIDS, including detailed information on HIV treatment and care and on the stages of HIV infection (in English and Spanish)
- The World Health Organization's 2010 antiretroviral therapy guidelines provide recommendations on when to initiate cART
- Information about Primo-SHM is available
- Patient stories about living with HIV/AIDS are available through Avert and through the charity website Healthtalkonline

## Introduction

The optimal management of primary HIV infection (PHI) and the possible impact of temporary combination antiretroviral therapy (cART) on clinical outcome are controversial [1],[2]. Reported benefits of temporary early cART from previous observational studies include lowering of the viral set point [3]–[5], a slower decline of CD4 T cells [6], partial normalization of CD4 T cell subsets [7], preservation of HIV-specific immune responses [8]–[10], and limitation of viral reservoirs established during the first few weeks after transmission [11]. However, other cohort studies have not confirmed an effect on viral set point [12]–[15] or have reported a similar or faster decline of CD4 T cells in patients treated during PHI [16],[17].

From a clinical perspective, an important question is whether patients who are treated during PHI remain off treatment longer than patients in whom treatment is deferred until indicated based on their CD4 cell count or clinical condition. A randomized controlled trial of 6 mo of zidovudine monotherapy in patients with PHI reported a reduction of minor opportunistic infections during the first year of follow-up [18]. Results of two randomized controlled trials in the cART era suggested a clinical benefit of temporary treatment during PHI [19],[20]. Preliminary results of the SPARTAC trial, which compared 12 and 48 wk of cART with no therapy during PHI, reported a modest delay in disease progression after 48 wk of cART [19]. The SETPOINT study aimed to compare 36 wk of cART with deferred therapy in early HIV infection, but was prematurely stopped in June 2009 by the Data Safety and Monitoring Board, because of a higher rate of disease progression in the untreated arm [20].

We conducted the Primo-SHM trial, in which patients with PHI were randomized between no treatment and 24 and 60 wk of early cART. The aim of the study was to assess the clinical benefit of temporary cART initiated during PHI, measured by the time that patients could remain off therapy until subsequent (re)start of cART was indicated based on current treatment guidelines, and to assess the optimal duration of such early treatment.

## Methods

### Study Population

Inclusion criteria were age over 18 y and laboratory evidence of PHI infection, defined as a negative or indeterminate Western blot in combination with detectable plasma HIV-1 RNA (Fiebig stage I–IV) or, in case of a positive Western blot, a documented negative HIV screening test in the previous 180 d (Fiebig stage V–VI [21]). Women were counseled to use adequate contraception; pregnant and breast-feeding women were excluded. The study was approved by the Medical Ethics Committee of each participating site, and written informed consent was obtained from all participants. The study protocol and the CONSORT checklist are provided as Text S1 and Text S2, respectively.

### Design

The Primo-SHM trial was a multicenter, open-label randomized controlled trial comparing temporary early cART (24 or 60 wk) with no treatment. Patients were recruited in 13 HIV treatment centers in the Netherlands. Participants were randomly assigned to receive no treatment or 24 or 60 wk of cART (three-way randomization). In cases where treatment was clinically indicated based on severe clinical symptoms (e.g., HIV-related meningitis) or the patient insisted on starting early cART, participants were randomized over the 24- and 60-wk treatment arms only (two-way randomization).

The study was designed to evaluate (1) the effect of temporary treatment during PHI on the viral set point, defined as the plasma viral load (pVL) at 36 wk after randomization in the no treatment arm and at 36 wk after treatment interruption (TI) in the treatment arms, and (2) the total time that patients were off therapy after randomization (treatment-free period). The analyses of the viral set point and the total time off therapy were restricted to patients who were randomized over all three study arms. A second comparison evaluated the optimal duration of early cART by comparing all patients who were treated with 24 or 60 wk of cART, including patients from both the three- and two-way randomization.

Recruitment started 1 May 2003 and continued until 31 March 2010. The present analysis includes follow-up data until 14 September 2011.

### Randomization

Patients were allocated to one of the three study arms using a computerized minimization algorithm with stratification for the stage of PHI (Fiebig stage I–II, III–IV, or V–VI). The Dutch HIV Monitoring Foundation performed the randomization procedure, had no interaction with study participants, and was responsible for data management. Randomization results were sent by fax to the clinical investigators, who were unaware of the allocation procedure.

### Procedures

Early cART consisted of a triple-class regimen of zidovudine/lamivudine (300/150 mg bid), efavirenz (600 mg qd), and lopinavir/ritonavir capsules (533/133 mg bid). The last was discontinued when the pVL dropped below 50 copies/ml. Changes to this regimen were allowed in case of transmitted drug resistance or if one of the drugs was contraindicated or not tolerated. After 28 January 2008, zidovudine/lamivudine was replaced by tenofovir/emtricitabine (245/200 mg qd), according to the Dutch standards of care, and lopinavir/ritonavir tablets (600/150 mg bid) replaced the capsules. Patients on early cART were required to reach viral suppression below 50 copies/ml in plasma before interrupting therapy.

Baseline evaluations included a medical history including the presence of symptoms compatible with an acute retroviral syndrome, a physical examination, and collection of blood for routine hematology and chemistry, CD4 and CD8 cell counts, pVL, hepatitis B and C serology, and storage of peripheral blood mononuclear cells. All study sites used a sensitive HIV RNA assay with comparable accuracy and a lower limit of detection of 40 or 50 copies/ml [22]. The assays that were used were Amplicor HIV-1 Monitor ultrasensitive RNA assay (Roche), Amplicor HIV-1 Monitor, Cobas Amplicor, Cobas TaqMan HIV-1 (Roche Diagnostics), m2000rt HIV RNA (Abbott), NucliSens EasyQ (bioMérieux), Quantiplex bDNA 3.0 (Chiron), and Versant HIV RNA 3.0 Assay (Siemens Healthcare Diagnostics). Standard HIV-1 genotyping and subtyping were performed at most participating sites. Drug-resistance mutations were identified according to the World Health Organization Surveillance Drug-Resistance Mutation list [23], using the calibrated population resistance tool of the Stanford University HIV Drug Resistance Database (http://hivdb.stanford.edu/) [24]. In patients recruited at the Academic Medical Center of the University of Amsterdam, additional viral and host genetic analyses were performed: the presence of CXCR4-using viruses at baseline, CCR5 Δ32 genotyping, and human leucocyte antigen (HLA) typing. CXCR4 coreceptor usage was determined using the MT-2 assay [25].

Randomization was performed at week 0, and patients randomized to receive cART started treatment. Patients were scheduled for follow-up visits at weeks 2, 4, 8, and 12, and every 12 wk thereafter for the duration of the study. Additional visits were scheduled at weeks 4, 8, and 12 following TI. During each follow-up visit, blood was collected for routine hematology and chemistry, CD4 and CD8 cell counts, pVL, and storage of peripheral blood mononuclear cells.

### Primary End Points

The primary efficacy end points were (1) the viral set point, defined as pVL at 36 wk after randomization in the no treatment arm and pVL at 36 wk after TI in the treatment arms, and (2) the total time that patients were off therapy, defined as the time between randomization and start of cART in the no treatment arm, and as the time between TI and restart of cART in the treatment arms. We chose the viral set point as the pVL at 36 wk to allow for stabilization of the pVL in untreated and treated patients [3],[5],[13],[14] and to minimize drop out due to rapid disease progression. (Re)start of cART was defined as the moment when the criteria for (re)start of cART were met—a CD4 cell count below 350 cells/mm^3^ on two consecutive occasions, severe constitutional symptoms, or the occurrence of an AIDS-defining event [26]—or if the physician or patient insisted on (re)initiating cART, whichever occurred first. We also explored the time to (re)start of cART with a CD4 cell count threshold of 500 cells/mm^3^ or less on two consecutive occasions [27],[28]. In the no treatment arm, the CD4 count criterion was not applied during the first 12 wk after randomization, because of the transiently low CD4 cell counts often observed during PHI.

### Statistical Analysis

At the time the protocol was developed in 2002, insufficient data were available to make a reliable estimation of the sample size needed to detect a clinically significant difference between the study arms in viral set point and time to (re)start of cART. An estimated number of 30 patients were expected to be enrolled annually, and in the protocol amendment of 28 January 2008, the end of study inclusion was set at March 2010, when we expected to have enrolled approximately 200 patients.

As defined in the study protocol, we conducted a modified intention-to-treat (mITT) analysis: participants who withdrew consent because they insisted on starting early cART while randomized to the no treatment arm or those who declined therapy after being randomized to one of the treatment arms were excluded from the analysis. In a standard intention-to-treat analysis, patients who are randomized to the no treatment arm but insist on starting treatment immediately have 0 d of survival since they reach the study end point immediately at initiation of treatment, and patients who are randomized to one of the treatment arms but refuse to start early treatment reach the study end point only at the moment they eventually start cART. This results in a strong and unnecessary bias in favor of the treatment arms. Therefore, we report the mITT analysis, in which patients who did not start with the allocated treatment plan were excluded.

Participants who were lost to follow-up while still on early cART were not included in the analyses. Patients who discontinued early cART before completing the scheduled period of 24 or 60 wk remained in the mITT analysis. Participants who did not discontinue early cART at the scheduled TI date or were lost to follow-up after randomization/TI were considered having reached the (re)start end point. Per protocol analyses were performed excluding patients who discontinued cART earlier than planned and patients who did not discontinue cART at the scheduled TI date but instead elected to continue cART for reasons other than a low CD4 count or symptomatic disease. Patients who had not yet (re)started cART were censored at the last follow-up visit.

Reported results concern data from the three-way randomized patients unless stated otherwise. Viral set point and the CD4 cell count measured at viral set point were compared using one-way ANOVA. The evolution of pVL and CD4+ T cell count following randomization/TI in the no treatment/treatment arms were analyzed using linear mixed models incorporating repeated measurements. Both the pVL and CD4 evolution showed a tri-phasic pattern with distinct slopes from week 0 to 8, week 8 to 36, and week 36 to 144. The total time off therapy for the no treatment versus the treatment arms was analyzed using the mITT population of the three-way randomized patients. For the second comparison, which evaluated the optimal duration of early cART, we combined the data of all treated patients, including those participants who were randomized over the two treatment arms. In both analyses, the total time off therapy was compared across the study arms using Kaplan-Meier plots and log rank tests.

We fitted a series of multivariable Cox regression models of time to (re)start of cART using the mITT population and the per protocol population of the three-way randomized patients separately and of the three- and two-way randomized patients combined. Because the sample size of our study was relatively small, the study arms might by chance be imbalanced with regard to certain prognostic factors. To reduce potential bias, we weighted all Cox regression analyses using propensity score weights [29]. Weights were calculated separately for each analysis using multivariable logistic regression with a generalized logit link function. The models included potential confounders that from the literature are known to influence HIV disease progression and that were measured at baseline: gender, age, country of origin, HIV-transmission risk category, baseline CD4 cell count, stage of PHI, symptomatic during PHI, resistance profile, HIV-1 subtype B/non-B, protective HLA alleles, X4/R5-tropism, heterozygosity for CCR5 Δ32 deletions, and viral hepatitis. All models using data from the three- and two-way randomized patients combined were additionally adjusted for the randomization scheme of each patient. The proportional hazards assumption was checked.

We hypothesized that the proposed mechanism by which early cART may result in a clinical benefit was by lowering of the viral set point and/or by increasing the CD4 cell count during the treatment period. Therefore, we also fitted Cox models that included the viral set point at 36 wk after randomization/TI in the no treatment/treatment arms and the CD4 cell count measured at viral set point. To further explore which factors might be associated with time to (re)start of cART, other than temporary early cART, viral set point, and CD4 cell count at viral set point, we performed a final sub-group analysis for which we selected all patients who had been randomized to one of the early treatment arms and fitted a multivariable Cox model using a stepwise selection process using all aforementioned variables. Data were analyzed using SPSS version 18.0 (SPSS) and SAS version 9.1.3 (SAS Institute). All reported *p*-values were two-sided and considered statistically significant when less than 0.05.

## Results

### Patient Characteristics

**Figure 1:**
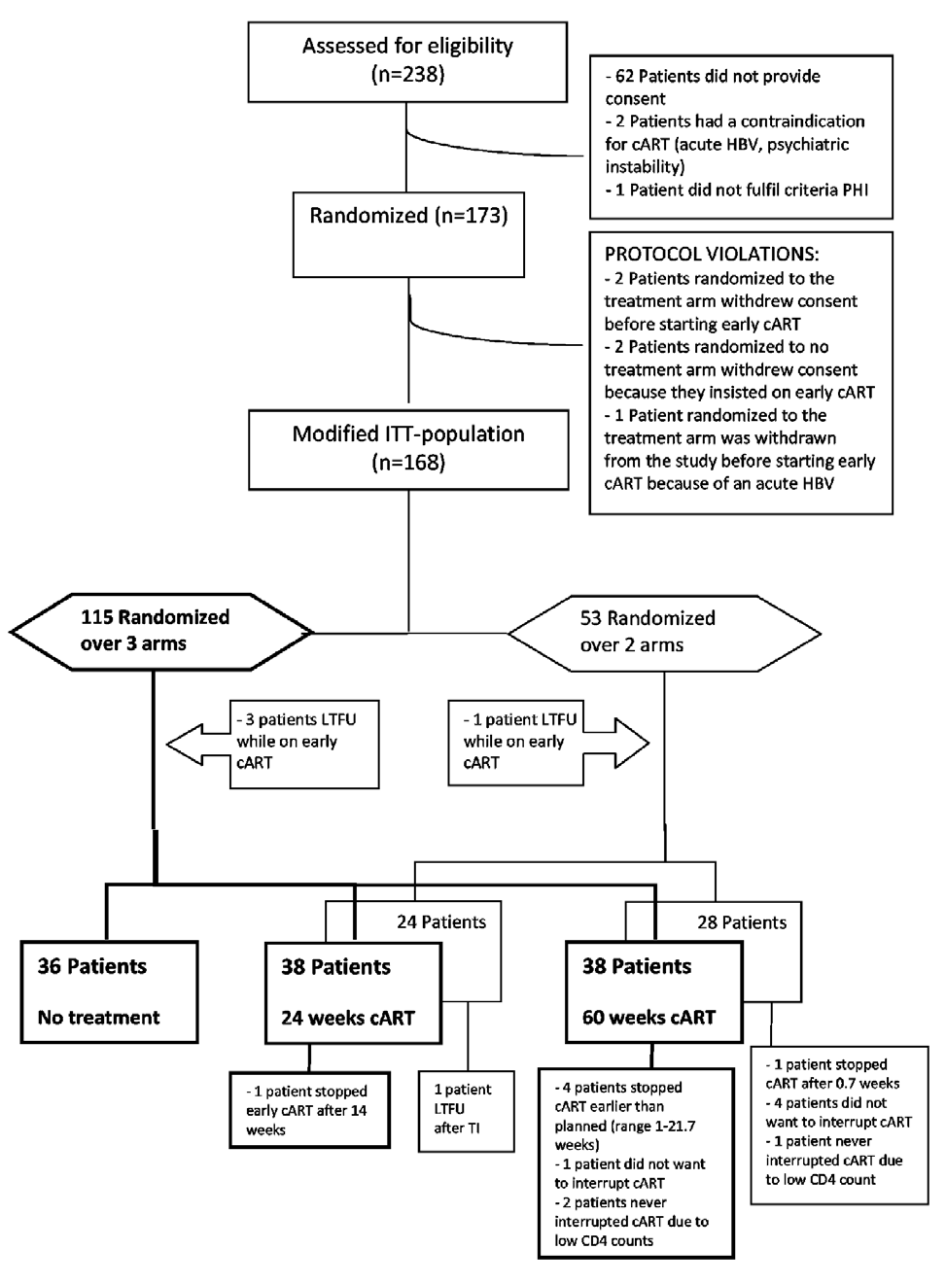
CONSORT flow diagram. HBV, hepatitis B virus; LTFU, loss to follow-up.

The patient enrollment is summarized in Figure 1: 238 eligible PHI patients were screened, of whom 173 were enrolled. Four participants withdrew consent and one patient developed an acute hepatitis B infection and was withdrawn from the study by the treating physician immediately following randomization. These five patients were excluded from all analyses. The mITT population consisted of 168 patients: 115 were randomized over the three study arms, and 53 were randomized over the two treatment arms only. Four patients were lost to follow-up while on early cART and were not included in the survival analyses. Of the three-way randomized patients, five interrupted cART earlier than planned, and three did not interrupt cART as planned, of whom two had a low CD4 cell count at the scheduled TI date. Of the two-way randomized participants, one interrupted cART earlier than planned, and five participants did not discontinue early cART as planned, of whom one had a low CD4 cell count at the scheduled TI date. These 14 patients remained, according to the protocol, in the analysis (Figure 1). Participants were followed for a period between 3 and 400 wk, with a median follow-up of 100 (interquartile range [IQR] 60–160) wk.

Demographic and baseline characteristics of the three-way randomized patients are summarized in Table 1. Most patients were men who have sex with men, had a negative or indeterminate Western blot (Fiebig stage I–IV), and were symptomatic during PHI. The mean baseline CD4 cell count was 458 (standard deviation [SD] 183) cells/mm^3^ in the no treatment arm, and 584 (230) cells/mm^3^ and 483 (241) cells/mm^3^ in the 24- and 60-wk treatment arms. The multivariable Cox regression analyses were adjusted for this baseline difference. Nine of 99 patients (9%) for whom HIV genotyping was available harbored transmitted drug-resistance mutations: in eight patients a single mutation in reverse transcriptase or protease was present, and one patient harbored a mutation in both reverse transcriptase and protease. All participants were treated with at least three active drugs during early cART. The presence of CXCR4-using viruses at baseline, CCR5 Δ32 heterozygosity, and HLA B27 or B57 were equally distributed between the three study arms. One untreated patient had a chronic hepatitis B infection, and none had hepatitis C infection. Patients on early cART had a median delay of 2 (IQR 0–6) d between randomization and start of early cART. The study drugs were well tolerated and caused no serious adverse events.

**Table 1:** Baseline characteristics of 115 patients randomized over three study arms.

Characteristic	Study Arm		
	No Treatment (*n* = 36)	24 wk of cART (*n* = 40)	60 wk of cART (*n* = 39)
**Age (years), mean (SD)**	42 (11)	40 (10)	39 (9)
**Male**	36 (100)	36 (90)	38 (97)
**MSM**	31 (86)	31 (78)	34 (87)
**Born in the Netherlands**	33 (92)	33 (83)	32 (82)
**Stage of PHI**			
Fiebig I–IV	27 (75)	27 (68)	30 (77)
Fiebig V–VI	9 (25)	13 (32)	9 (23)
**Acute retroviral syndrome**	30 (83)	31 (78)	34 (87)
**CD4 cell count (cells/mm^3^), mean (SD)**	458 (183)	584 (230)	483 (241)
**Plasma HIV-1 RNA (log_10_ copies/ml), mean (SD)**	5.1 (0.9)	5.1 (0.9)	4.9 (0.9)
**Genotypic resistance mutations** a	1 (3)	5 (15)	3 (9)
**Subtype B virus** a	29 (91)	30 (91)	31 (91)
**HLA B27 or B57** b	1 (5)	1 (5)	1 (4)
**CCR5 Δ32 heterozygosity** c	1 (8)	3 (14)	5 (29)
**CXCR4-using virus** d	0 (0)	0 (0)	1 (6)
**Interval between diagnosis and randomization (weeks), median (IQR)**	4 (2–6)	4 (3–6)	4 (2–6)
**Early cART nucleoside backbone**			
Zidovudine/lamivudine	n.a.	19 (48)	22 (56)
Tenofovir/emtricitabine	n.a.	21 (52)	17 (44)

Data are *n* (percent) unless indicated otherwise.

a 16 patients with missing data.

b 51 patients with missing data.

c 65 patients with missing data.

d 60 patients with missing data.

MSM, men who have sex with men; n.a., not applicable.

No differences in baseline characteristics were seen between the three- and two-way randomized treated patients, with the exception of the nucleoside backbone (zidovudine/lamivudine versus tenofovir/emtricitabine) used during early cART (Table S1).

### Treatment Interruption, Viral Set Point, and CD4 Cell Count Changes

Of the three-way randomized patients, 66/76 treated participants (87%) had a pVL below 50 copies/ml at the time of TI. Five patients interrupted early cART on the scheduled TI date even though their pVL was not below 50 copies/ml (median pVL 161 [range 74–262] copies/ml). The remaining five participants had interrupted early cART before the scheduled TI date and had a median pVL of 977 (range 95–100,000) copies/ml.

**Figure 2:**
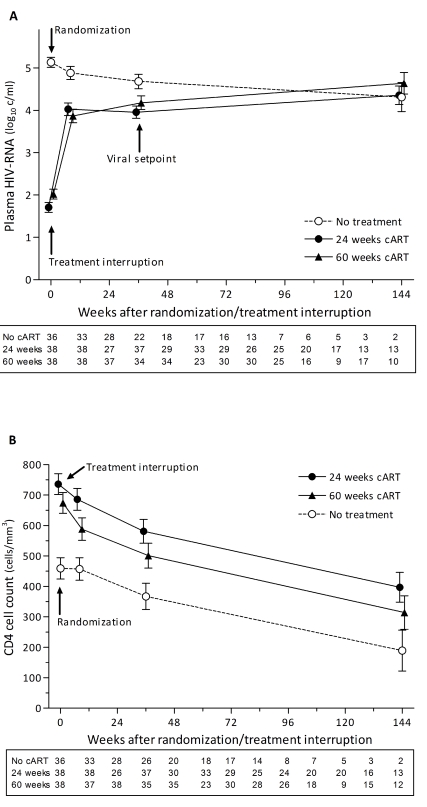
Plasma viral load and CD4 cell count after randomization/treatment interruption in the no treatment and treatment arms. Modeled mean pVL (A) and CD4 cell count (B) over time after randomization/TI in the no treatment and the 24- and 60-wk treatment arms for the group of patients randomized over the three study arms. Graphs show the estimates (± standard error of the mean) from the linear mixed models. The box below each graph shows the number of pVL and CD4 cell count measurements at each time point used for fitting the linear mixed models. c/ml, copies/ml.

Twelve weeks after TI, 69/71 participants (97%) for whom a pVL was available had a viral rebound. Following TI, the pVL rebounded during the first 8 wk, with an estimated increase of 0.29 and 0.23 log_10_ copies/ml/wk in the 24- and 60-wk treatment arms, respectively (*p* = 0.05). From week 8 to 36 after randomization/TI the pVL did not change significantly in the no treatment and 24-wk treatment arms, but increased by 0.01 log_10_ copies/ml/wk in the 60-wk treatment arm (*p* = 0.02; Figure 2A). The mean viral set point, 36 wk after randomization/TI, was 4.8 (SD 0.6) log_10_ copies/ml in the no treatment arm, and 4.0 (1.0) and 4.3 (0.9) log_10_ copies/ml in the 24- and 60-wk treatment arms, respectively (comparing all three study arms, *p*<0.001). Seven patients in the 24- and 60-wk treatment arms had a pVL<1,000 copies/ml at viral set point, of whom four had a pVL below 100 copies/ml, as compared to none in the no treatment arm. From week 36 to 144 the pVL increased by 0.18 and 0.21 log_10_ copies/ml/y (*p* = 0.9) in the 24- and 60-wk treatment arms until the pVL in the three study arms slowly converged 2–3 y after TI.

The mean CD4 cell count at TI in the 24- and 60-wk treatment arms was 737 (SD 245) and 672 (245) cells/mm^3^, respectively (*p* = 0.3). After TI the CD4 cell counts showed an initial rapid decline during the first 8 wk, with an estimated loss of 6.3 and 10.7 cells/mm^3^/wk in the 24- and 60-wk treatment arms, respectively (*p* = 0.3). From week 8 to 36 and week 36 to 144 after randomization/TI, the CD4 count decline was similar between all three study arms (*p* = 0.9 and *p* = 1.0 for both periods respectively; Figure 2B). The mean CD4 count at viral set point was 383 (SD 158) cells/mm^3^ in the no treatment arm, and 584 (202) and 503 (254) cells/mm^3^ in the 24- and 60-wk treatment arms, respectively (comparing all three study arms, *p*<0.001).

### Total Time Off Therapy

**Figure 3:**
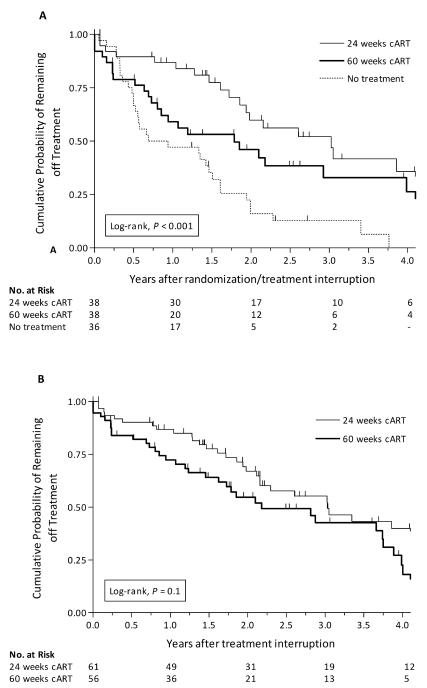
Probability of remaining off treatment in the no treatment and treatment arms. Kaplan-Meier curves of the probability of remaining off treatment for the no treatment arm versus the 24- and 60-wk treatment arms in the patients randomized over the three study arms using the mITT population (A), and for the 24- versus 60-wk treatment arms, including both treated patients randomized over all three study arms and patients randomized over the two treatment arms, using the per protocol population (B).

At the time of analysis, 32/36 patients (89%) in the no treatment arm had initiated cART, while 22/38 (58%) in the 24-wk treatment arm and 24/38 (63%) in the 60-wk treatment arm had restarted cART (*p* = 0.008). 54/78 participants (69%) (re)started cART according to the CD4 count criteria, four patients (5%) because of severe symptoms/AIDS diagnosis, and 20 (26%) patients because of a preference by physician or patient to restart cART. Of note, 14 of these 20 patients had already had one CD4 count <350 cells/mm^3^. The mean CD4 cell count at (re)start was 294 (SD 126) cells/mm^3^ in the no treatment arm, and 322 (114) and 317 (127) cells/mm^3^ in the 24- and 60-wk treatment arms (*p* = 0.7). The median time off therapy after randomization/TI was 0.7 (95% confidence interval [CI] 0.0–1.8) y in the no treatment arm, and 3.0 (1.9–4.2) and 1.8 (0.5–3.0) y in the 24- and 60-wk treatment arms, respectively (log rank test comparing all three study arms, *p*<0.001; Figure 3A). Using a CD4 cell count threshold of 500 instead of 350 cells/mm^3^ for (re)start of cART, 34/36 (94%) patients in the no treatment arm, 26/38 (68%) in the 24-wk treatment arm, and 27/38 (71%) in the 60-wk treatment arm would have (re)initiated cART (*p* = 0.01). The median time off therapy after randomization/TI would have been 0.5 (95% CI 0.4–0.6) y in the no treatment arm, and 2.0 (1.2–2.9) and 0.7 (0.3–1.0) y in the 24- and 60-wk treatment arms, respectively (log rank test, *p* = 0.002).

Combining all treated patients (*n* = 128), including those participants who were randomized over the two treatment arms, the median time off therapy was 3.1 (95% CI 2.1–4.0) and 2.1 (1.1–3.1) y in the patients treated for 24- and 60-wk, respectively (*p* = 0.03). However, 6/128 patients discontinued cART earlier than planned, and 5/128 did not discontinue cART at the scheduled TI date even though their CD4 cell counts were high (range 480–863 cells/mm^3^; Figure 1). Excluding these 11 patients in the per protocol analysis, the median time off therapy was 3.0 (95% CI 2.1–3.9) and 2.2 (1.1–3.3) y in the patients treated for 24- and 60-wk, respectively (log rank test, *p* = 0.1; Figure 3B).

### Factors Associated with Time to (Re)Start of cART

We fitted six Cox regression models to compare the time to (re)start of cART between the patients in the no treatment and 24- and 60-wk treatment arms (Table 2). To account for potential bias, including the differences in baseline CD4 cell count between the three study arms, all Cox models were adjusted for differences between groups in potential confounding factors by propensity score weighting. In the adjusted Cox model for the mITT population of the three-way randomized patients, the hazard ratio for (re)start was significantly smaller for both treatment arms compared to the no treatment arm (Model 1; Table 2). There were no significant differences between the 24- and 60-wk treatment arms (*p* = 0.45). Repeating the analysis for the per protocol population of the three-way randomized patients yielded similar results (Model 2). Then we pooled the three-way and two-way randomized patients and performed Cox analyses for both the mITT population and the per protocol population, adjusting both analyses for the randomization scheme of each patient. The hazard ratio for time to (re)start of cART was significantly smaller in both treatment arms as compared to the no treatment arm (Models 3 and 4), and there were no significant differences between the two treatment arms (*p* = 0.77 and *p* = 0.66, respectively).

**Table 2:** Propensity-score-weighted Cox proportional hazard models for time to (re)start of cART.

Characteristic	Model					
	1	2	3	4	5	6
**Patient population**	mITT	Per Protocol^a^	mITT	Per Protocol^a^	mITT	mITT
**Randomization scheme**	Three-way	Three-way	Three- and two-way	Three- and two-way	Three-way	Three- and two-way
**Number of patients**	112	106	164	153	112	164
**Fitted Cox model**						
24 wk cART^b^	0.42 (0.25–0.73), *p* = 0.002	0.43 (0.25–0.75), *p* = 0.003	0.42 (0.24–0.74), *p* = 0.003	0.41 (0.24–0.72), *p* = 0.002	0.77 (0.41–1.42), *p* = 0.40	0.82 (0.46–1.47), *p* = 0.50
60 wk cART^b^	0.55 (0.32–0.95), *p* = 0.032	0.56 (0.32–0.99), *p* = 0.047	0.45 (0.26–0.80), *p* = 0.006	0.47 (0.26–0.84), *p* = 0.011	0.78 (0.44–1.37), *p* = 0.38	0.56 (0.32–0.99), *p* = 0.045
Comparison of 24- and 60-wk cART	*p* = 0.45	*p* = 0.49	*p* = 0.77	*p* = 0.66	—	—
CD4 cell count at viral set point^c^	—	—	—	—	0.42 (0.32–0.53), *p*<0.001	0.50 (0.41–0.60), *p*<0.001
Viral set point^d^	—	—	—	—	1.49 (1.01–2.22), *p* = 0.047	1.87 (1.33–2.63), *p*<0.001

a Per protocol analysis excludes patients who discontinued cART earlier than planned and patients who did not interrupt cART but instead elected to continue cART for reasons other than a low CD4 count or symptomatic disease.

b Hazard ratio (95% CI) for early cART; the reference group is the no treatment arm.

c Hazard ratio (95% CI) per 100 CD4 cells/mm^3^ increase.

d Hazard ratio (95% CI) per 1 log_10_ copies/ml increase.

To explore the contribution of the lowering of the viral set point and the increase in CD4 cell count during early treatment to the timing of (re)start of cART, we added these two parameters to the Cox models. Both the viral set point and the CD4 count at set point were strong and independent predictors of time to (re)start of cART in both the three-way randomized patients and the three- and two-way randomized patients combined (Models 5 and 6). In these two models, early cART was no longer significantly associated with time to restart of cART, suggesting that the lowering of the viral set point and the increase of the CD4 cell count during the early treatment period explained for the most part why early cART resulted in a clinical benefit.

In an attempt to identify further factors that predict a longer time to restart of cART, we performed a final sub-group analysis of all patients who had been treated with temporary early cART. This model included both the viral set point and CD4 count at set point. We found that the stage of PHI, the self-reported occurrence of an acute retroviral syndrome, baseline pVL, and all other investigated factors were not statistically significantly associated with time to (re)start of cART.

## Discussion

The present randomized trial provides the strongest evidence to date of a clinical benefit of temporary cART initiated during PHI. Early cART transiently lowered the viral set point by 0.5–0.8 log_10_ copies/ml, increased the CD4 cell count, and deferred the need for initiation of cART during chronic HIV infection by 1.1–2.3 y. Both viral set point and CD4 cell count measured at viral set point were associated with time to (re)start of cART. The duration of temporary early cART was not predictive, suggesting that 24 wk of cART would be sufficient. The time off therapy was longer in the 24-wk treatment arm than in the 60-wk treatment arm, but in the per protocol analysis, and in the Cox models, which were adjusted for possible confounders, there was no statistically significant difference between the 24- and 60-wk treatment arms.

Overall, these findings are in agreement with the data of the SETPOINT study and SPARTAC trial, in which, respectively, 36 and 48 wk of cART during early HIV infection modestly delayed the need for subsequent initiation of cART [19],[20].

An important strength of our study is that it showed a significant benefit of temporary early cART as compared to deferred therapy even using conservative mITT analyses, in which patients who discontinued cART earlier than planned remained in the analyses and those who did not discontinue cART at the scheduled TI date were considered as having reached the study end point.

The study has several limitations. First, despite the random allocation of patients to the different study arms, the mean baseline CD4 cell count was by chance higher in the 24-wk treatment arm, which might have affected the time to restart in this group. However, the mean baseline CD4 cell count was not different between the no treatment and 60-wk treatment arms, whereas the time off therapy was significantly longer in the patients treated for 60 wk (*p* = 0.02). More importantly, after adjusting for baseline CD4 cell count in the Cox models, temporary early cART remained associated with a longer time to subsequent reinitiation of cART. Second, by providing the option of randomizing patients over the two treatment arms only, we might have introduced selection bias, as patients with more severe symptoms might have been less likely to be enrolled in the three-way randomization. However, we observed that the baseline characteristics, viral set point, CD4 cell count measured at viral set point, and the time to (re)start of cART did not differ between the three- and two-way randomized treated patients. Additionally, the adjusted Cox models also showed that the time to (re)start of cART was not different for the treated patients randomized over three or two study arms. Third, according to current treatment guidelines zidovudine/lamivudine was replaced by tenofovir/emtricitabine halfway through the trial, which is a potential source of variability in baseline characteristics between treatment groups. However, the proportion of PHI patients treated with both drugs was comparable in both treatment arms (Table 1).

The rate of HIV disease progression in our study was high: the median time off therapy in the no treatment arm was less than 1 y. More than 80% of patients presented with an acute retroviral syndrome, which is a strong predictor of HIV disease progression [30], suggesting that our results may not be generalizable to asymptomatic seroconverters. Our findings are consistent with a German cohort study in which 56 patients with untreated PHI had a median time to CD4 cell count <350 cells/mm^3^ of 8.3 mo after seroconversion [31]. Data from the CASCADE cohort, a collaboration of international cohorts of patients with a well-estimated date of HIV seroconversion [32], demonstrated that in 179 untreated seroconverters the median time to initiation of cART or reaching a CD4 cell count below 350 cells/mm^3^ was approximately 1.5 y [6]. However, it was not reported whether patients were symptomatic or not during the acute stage of the disease.

The stage of PHI was not associated with time to (re)start of cART. This is in contrast with findings from two previous cohort studies. In one study, initiation of cART within 2 wk of antibody seroconversion was associated with viral and immunological benefits during at least 24 wk after TI as compared to starting therapy between 2 wk and 6 mo after HIV seroconversion [3]. The second study reported that if early cART was initiated within 60 d after estimated HIV infection, it resulted in reduced pVL and proviral HIV DNA levels as compared to later starters (between 61 and 120 d) or untreated controls for more than 1 y after TI [33]. The treated patients in our study had a median delay of 5 (IQR 3–7) wk between the diagnosis of PHI and the start of early cART, which means that the golden hour, in which the greatest benefits of early cART could have been achieved, was possibly already missed. Nevertheless, even with this delay we observed a clear benefit of initiating cART during PHI.

Temporary cART initiated during PHI deferred the subsequent restart of cART, which, according to the Cox models, was most likely caused by the effects of the CD4 gain during treatment and the transient lowering of the viral set point. The question remains how the latter might be explained. We know that two critical events in PHI are the massive destruction of CD4 T cells in the gastrointestinal tract and the establishment of latent HIV reservoirs. Early treatment may result in viral suppression and immune restoration in gut-associated lymphoid tissue [34]. Alternatively, it may have an effect on the cellular HIV proviral load and limit the size of the viral reservoirs [35],[36]. Others have postulated that early treatment enables virus-specific CD8+ T cells to mature into fully differentiated effector cells, which might be important in viral control [37]. Early treatment is also suggested to help preserve specific anti-HIV responses [9],[38], though we previously found that HIV-specific CD4 T cell responses provided no explanation for the lower viral set point in patients treated during PHI [39]. Recently, we reported that HIV-1 dual infection (coinfection or superinfection) was the main factor associated with CD4 cell count decline in a cohort of 37 untreated men with subtype B PHI [40]. A potential benefit of temporary early cART may therefore be the prevention of early HIV-1 superinfection.

The gain in treatment-free years must be weighed against potential disadvantages of starting cART in the acute stage of the disease, a period in which patients are often physically and emotionally distressed and adherence may be suboptimal. In addition, early cART is often initiated before baseline resistance test results are available and therefore may require “overtreatment” to ensure an effective drug regimen, which may increase the risk of drug toxicity. In our study, patients were started on a triple-class therapy. In any case, if early treatment is considered, it should at least include a boosted protease inhibitor until resistance testing results are available [1]. Another concern may be the risk of developing drug resistance after TI. Extended follow-up studies will be needed to address this question.

Structured TI studies in chronic HIV infection have fallen into disfavor after the SMART trial demonstrated that CD4-guided TI was associated with adverse outcomes and a rapid CD4 cell count decline as compared to continuous therapy [41]. The question is whether this also holds true for patients who have initiated cART shortly after seroconversion, before the development of severe immunological dysfunction [42]. The ANRS PRIMO cohort reported that a larger increase in CD4 cells during early cART was associated with a markedly steeper decline after TI, and the benefit of a limited course of cART was questioned [17]. In our study, after an initial rapid, but limited CD4 cell decline during the first 8 wk following TI in both the 24- and 60-wk treatment arms, the slope of CD4 cell decline was comparable between all three study arms.

Nonetheless, even in the patients in our study who received temporary early cART, the total time off therapy was relatively short. The lowering of the viral set point was transient, suggesting loss of protective immune functions and the emergence of viral escape mutants. Therefore, a reasonable question is whether early cART should not be interrupted but continued for life, given the concern that uncontrolled HIV replication and chronic immune activation carry an increased risk of morbidity and mortality at all stages of HIV infection [27]. Additionally, the continuation of cART may have a public health benefit as it decreases infectiousness [43]–[45].

In conclusion, this randomized study demonstrates a clear clinical benefit of temporary cART initiated during PHI. Early cART transiently lowered the viral set point and deferred the need for restart of cART during chronic HIV infection. Although extended follow-up studies are needed to evaluate the long-term benefits of such early treatment, starting cART when the patient is ready to do so seems the most reasonable advice for patients with PHI.

## Supporting Information

Table S1 Baseline characteristics, viral set point, and CD4 cell count measured at viral set point of the three- and two-way randomized treated patients.
(DOC)

Text S1 Trial protocol.
(DOC)

Text S2 CONSORT checklist.
(DOC)

## Abbreviations

cART: combination antiretroviral therapy
CI: confidence interval
HLA: human leucocyte antigen
IQR: interquartile range
mITT: modified intention-to-treat
PHI: primary HIV infection
pVL: plasma viral load
SD: standard deviation
TI: treatment interruption
