# Supplementary material for: No Treatment versus 24 or 60 Weeks of Antiretroviral Treatment during Primary HIV Infection: The Randomized Primo-SHM Trial
Source: PLoS Med. 2012 Mar 27;9(3):e1001196. doi: 10.1371/journal.pmed.1001196 (PMC3313945; doi:10.1371/journal.pmed.1001196)
Supplement: Table S1 — Baseline characteristics, viral set point, and CD4 cell count measured at viral set point of the three- and two-way randomized treated patients. (DOC) [file pmed.1001196.s001.doc]

**Table S1.** Baseline characteristics, viral setpoint and CD4 cell count measured at viral setpoint of the three- and two-way randomized treated patients.

| **Characteristic** | **3-way randomized patients (*n* = 79)** | **2-way randomized patients (n = 53)** | ***P*-value^†^** |
| --- | --- | --- | --- |
| **Age (years), mean (SD)** | 39 (10) | 37 (9) | 0.1 |
| Male | 74 (94) | 48 (91) | 0.5 |
| **MSM** | 65 (82) | 46 (87) | 0.5 |
| **Born in the Netherlands** | 65 (82) | 47 (89) | 0.3 |
| **Stage of PHI** |  |  |  |
| Fiebig I-IV | 57 (72) | 40 (75) | 0.7 |
| Fiebig V-VI | 22 (28) | 13 (25) |
| **Acute retroviral syndrome** | 65 (82) | 45 (85) | 0.7 |
| **CD4 cell count^a^ (cells/mm^3^),**  **mean (SD)** | 534 (239) | 573 (246) | 0.4 |
| **Plasma HIV-1 RNA^a^ (log_10_ copies/ml),**  **mean (SD)** | 5.0 (0.9) | 4.9 (1.1) | 0.6 |
| **Genotypic resistance mutations^b^** | 8 (12) | 4 (9) | 0.8 |
| **Subtype B virus^b^** | 61 (91) | 37 (84) | 0.3 |
| **HLA B27 or B57^c^** | 2 (7) | 2 (4) | 1.0 |
| **CCR5∆32 heterozygousity^d^** | 8 (21) | 4 (18) | 1.0 |
| **CXCR4-using virus^e^** | 1 (3) | 1 (5) | 1.0 |
| **Interval between diagnosis and randomization (weeks), median (IQR)** | 4 (3-6) | 4 (3-7) | 0.8 |
| **Early cART nucleoside backbone** |  |  |  |
| zidovudine/lamivudine | 41 (52) | 38 (72) | 0.02 |
| tenofovir/emtricitabine | 38 (48) | 15 (28) |
| **CD4 cell count at viral setpoint** (cells/mm^3^), mean (SD)^f^ | 544 (231) | 629 (270) | 0.07 |
| **Viral setpoint** (log_10_ copies/ml), mean (SD)^f^ | 4.1 (1.0) | 4.3 (0.9) | 0.4 |

Data are n (percent) unless indicated otherwise.

† P-value based on Student’s t-test or the Mann-Whitney test for continuous variables and χ^2^ or Fisher's exact tests for proportions.

**^a^**1 patient with missing data.

**^b^**21 patients with missing data.

**^c^**56 patients with missing data.

^§^72 patients with missing data.

^e^79 patients with missing data.

^f^7 patients with missing data.

MSM, men who have sex with men.
