# Supplementary material for: No Treatment versus 24 or 60 Weeks of Antiretroviral Treatment during Primary HIV Infection: The Randomized Primo-SHM Trial
Source: PLoS Med. 2012 Mar 27;9(3):e1001196. doi: 10.1371/journal.pmed.1001196 (PMC3313945; doi:10.1371/journal.pmed.1001196)
Supplement: Text S1 — Trial protocol. (DOC) [file pmed.1001196.s002.doc]

#### **Protocol**

#### **Primo-SHM**

**A randomized, triple-arm study to compare the viral and immunological outcome of HAART during 6 versus 15 months versus no therapy in patients with primary HIV‑1 infection**

**Jan M. Prins**

**Radjin Steingrover**

**Joep M.A. Lange**

**Dpt of Medicine, Division of Infectious Diseases, Tropical Medicine and AIDS,**

**Academic Medical Center, Amsterdam, the Netherlands**

**Frank de Wolf**

**HIV Monitoring Foundation (SHM)**

**Amsterdam, The Netherlands**

**Suzanne Jurriaans**

**Georgios Pollakis**

**Dpt of Medical Microbiology**

**Academic Medical Center, Amsterdam, the Netherlands**

**Hanneke Schuitemaker**

**Dpt of Experimental Immunology**

**Academic Medical Center, Amsterdam, The Netherlands**

**Frank Miedema**

**Dpt of Immunology, University Medical Center Utrecht**

**Utrecht, the Netherlands**

#### **Rationale**

#### Although no consensus exists, evidence is accumulating that early treatment with highly active antiretroviral therapy (HAART) in patients who present with an acute or primary HIV infection (PHI) has a beneficial effect on early and late disease progression^1-3^. In early HIV infection, treatment with HAART may improve the initial cellular response and protect naïve CD4^+^ cells against infection and thus enhance the immune response^4-6^. A treatment period during PHI could lower the viral ‘set-point’, characteristic of chronic HIV infection. A lower viral set-point during chronic HIV-infection in its turn is correlated with a better long term clinical outcome. However, the benefits of therapy during PHI have never been demonstrated in clinical trials so far. The optimal duration of such a treatment period during PHI also remains unknown. The lack of evidence regarding the optimal strategy during PHI warrants randomised, controlled investigation. Moreover, the decision whether or not to treat PHI should preferably be placed within the setting of a clinical trial.

#### The aim of this study is to provide data on the effect of treatment during PHI on the viral set-point and the optimal duration of such treatment.

Design

This will be an open-label, semi-factorial, randomized, triple-arm study comparing no treatment versus the use of a quadruple HAART regimen during 6 versus 15 months in patients with a primary HIV-1 infection

The study will enrol patients from physicians who participate in the Dutch HIV Monitoring Foundation (Stichting HIV Monitoring (SHM), formerly known as Athena). The aim of the study is to enrol all patients who meet the inclusion criteria, thus the number of patients is not predetermined. An estimated number of 30 patients will be expected to enrol annually.

### Data from patients who meet the inclusion criteria but refuse therapy will be collected in the same way as data collected from patients on treatment. In this way, these patients form a second reference group of untreated patients. Enrolled patients who discontinue HAART before completing the period of 6 or 15 months will remain in the study and they will be sampled in the same fashion as the other patients.

For patients that need to start therapy, randomization will be limited to the two treatment groups exclusively.

(See flowchart on next page)

###

**Primary objectives**

To evaluate the effect of 6 or 15 months of treatment with a quadruple combination of HAART versus no treatment in PHI. Efficacy will be evaluated by:

1. the viral set-point reached for the three groups and viral rebound kinetics after treatment discontinuation for the two treatment groups.
2. the time that patients can remain off therapy after treatment discontinuation.

**Secondary objectives**

To evaluate:

1. the effect of treatment on plasma viral load and CD4^+^ T cells
2. the effect of no treatment versus a treatment duration of 6 versus 15 months on the kinetics of HIV-1 specific CD4^+^ and CD8^+^ T-cell responses and their state of maturation, activation and proliferative capacity.
3. the development and efficacy of the humoral immune response.
4. the quality of life.

Inclusion criteria

1. Diagnosis of acute/ recent HIV-1 infection: plasma HIV-1 RNA load detectable and/or detectable serum p24 antigen *and* one of the following:

A ELISA: HIV-1 specific antibodies negative, *or*

B ELISA: low level antibodies or HIV-1 specific antibodies positive

and a negative, incomplete or indeterminate Western Blot (antibodies against a maximum of three of the HIV specific proteins), *or*

C ELISA: HIV-1 specific antibodies positive and positive Western Blot, but with documented negative HIV-1 ELISA in the preceding 180 days.

N.B. Patients should preferably start treatment as soon as possible after diagnosis of acute/ recent HIV-1 infection, but in any case within 180 days after diagnosis.

1. Written informed consent, including permission to store samples.
2. Patient is at least 18 years of age.
3. For female subjects: the use of adequate contraception

**Exclusion criteria**

1. Previous test result with HIV-1 RNA detectable or P24 antigen positive > 180 days before presentation
2. For female subjects: pregnancy (positive urine pregnancy test) or breast feeding

###### Study drugs and dosages

Subjects randomized to treatment will receive 6 or 15 months of the following regimen:

- emtricitabine/tenofovir (Truvada) 200/245 mg once daily
- efavirenz (Stocrin)* 600 mg once daily
- lopinavir/ritonavir (Kaletra) 600/150 mg b.i.d

N.B. After one measurement of HIV-RNA load < 50 copies/ml, Kaletra is to be discontinued.

**Follow up and measurements**

## Screening for adverse effects, physical examination, including vital signs*

### Quality of life assessments by questionnaire

Laboratory tests for safety:

- Hematology, including full blood count (FBC) and leukocyte differentiation*
- Chemistry including liver function test (LFT)*
- Urine analysis, including 24-hrs sample**
- Dual-energy X-ray absorptiometry (DEXA)**
- Markers of bone metabolism, incl. parathyroid hormone**
- Fasting plasma lipids, incl. hs-CRP, apoA1, apoB, insuline**

Virology:

- HIV-1 RNA load in plasma*
- Western blot (baseline)*
- Genotypic resistance at screening*

Immunology:

- T-cell subsets: absolute numbers of CD4/CD3 and CD8/CD3 positive cells (T lymphocytes), the CD4/CD8 ratio*
- Storage of PBMC’s, plasma and serum to perform additional assays
- HLA typing*

*) is part of routine patient care

**) AMC routine patient care

Sampling is to be performed at every patient visit (week , 2, 4, 8, 12, etc.). After treatment discontinuation: at week , 4, 8, 12, 24 and 36. For the no treatment and 6 month treatment groups, visits will be scheduled every 3 months until study completion at week 96 and every three months there after until patients (re)start HAART for chronic HIV infection.

Body material will be stored of all patients who provide appropriate informed consent, with the purpose to do additional investigations after the study has been completed. After the completion of the study, this material will be kept until these investigations have been done, with a maximum of five years. After a maximum duration of five years, these stored materials will be destroyed.

Analysis of the results

Analysis will include a comparison of the three arms (stratified for the patient subgroups A, B and C, see below) for the endpoints listed below. Analysis of the primary endpoint is scheduled for April 2012 based on two more years of enrollment and two years of follow-up after inclusion of the last patient.

Patients will be stratified in three subgroups, according to HIV-1 serology on first day of treatment:

Subgroup A: ELISA: HIV-1 specific antibodies negative.

Subgroup B: ELISA: low level antibodies or HIV-1 specific antibodies positive;

and a negative, incomplete or indeterminate Western Blot (antibodies against a maximum of three of the HIV specific proteins);

Subgroup C: ELISA: HIV-1 specific antibodies positive and positive Western Blot, but with documented negative HIV-1 ELISA in the preceding 180 days (patient subgroup C).

Primary endpoints:

1. Indication reached for start / reinitiation antiretroviral treatment:
   1. two times a CD4^+^  T cell count below 350 cells/μl during untreated follow-up
   2. the occurrence of an AIDS defining event
   3. symptomatic chronic HIV infection with severe constitutional symptoms
2. Time between diagnosis and start/reinitiation of HAART
3. Time between treatment discontinuation and reinitiation of HAART
4. Magnitude of viral set-point will be evaluated for both treatment groups 36 weeks after treatment discontinuation, and a comparison will be made between all groups at week 96 (= study end)

Secondary endpoints:

- CD4^+^ cell counts
- Comparison between all three groups of viral kinetics (including rebound) during the off-treatment periods
- Safety: (serious) adverse events, HIV related events and death
- Quality of life

In selected groups/individuals:

- HIV-1 specific CD4^+^ and CD8^+^ T-cell response and their state of activation and maturation
- Humoral immune response parameters

Visit schedule 6 month treatment group

| TRIAL PERIOD | Screening | Baseline |  |  |  |  | Interrupt |  |  |  |  |  |  |  |  |  |
| --- | --- | --- | --- | --- | --- | --- | --- | --- | --- | --- | --- | --- | --- | --- | --- | --- |
| WEEK | -4 TO –1 | 0 | 2 | 4 | 8 | 12 | 24 | 4 | 8 | 12 | 24 | 36 | 48 | 60 | 72 | Etc. ^††^ |
| Study duration (months) |  | 0 |  | 1 | 2 | 3 | 6 | 7 | 8 | 9 | 12 | 15 | 18 | 21 | 24 |  |
| Visit number | 1 | 2 | 3 | 4 | 5 | 6 | 7 | 1a | 2a | 3a | 4a | 5a | 6a | 7a | 8a |  |
| Eligibility criteria | X |  |  |  |  |  |  |  |  |  |  |  |  |  |  |  |
| Informed consent | X |  |  |  |  |  |  |  |  |  |  |  |  |  |  |  |
| Complete medical history | X |  |  |  |  |  |  |  |  |  |  |  |  |  |  |  |
| Prior antiretroviral therapy | X |  |  |  |  |  |  |  |  |  |  |  |  |  |  |  |
| Concomitant medications | X | X | X | X | X | X | X | X | X | X | X | X | X | X | X | X |
| Physical examination | X | X | X | X | X | X | X | X | X | X | X | X | X | X | X | X |
| Routine laboratory testing** | X | X | X | X | X | X | X | X | X | X | X | X | X | X | X | X |
| CD4^+^ / CD8^+^ lymphocytes | X | X | X | X | X | X | X | X | X | X | X | X | X | X | X | X |
| ELISA for HIV-1 antibody^†^ | X |  |  |  |  |  |  |  |  |  |  |  |  |  |  |  |
| Western blot^†^ | X |  |  |  |  |  |  |  |  |  |  |  |  |  |  |  |
| Genotypic resistance | X |  |  |  |  |  |  |  |  |  |  |  |  |  |  |  |
| Plasma HIV-1 RNA levels | X | X | X | X | X | X | X | X | X | X | X | X | X | X | X | X |
| Urine pregnancy test | X | X |  |  |  |  |  |  |  |  |  |  |  |  |  |  |
| Fasting plasma lipid spectrum* | X | X |  | X |  |  | X | X |  |  |  | X |  |  | X |  |
| Urine analysis* |  | X |  |  |  |  | X |  |  |  |  |  |  |  | X |  |
| Markers bone metabolism* |  | X |  |  |  |  | X |  |  |  |  |  |  |  | X |  |
| PBMC virology | X |  |  |  |  |  | X |  |  |  | X |  |  |  |  |  |
| PBMC/plasma/serum storage* |  | X | X | X | X | X | X | X | X | X |  | X | X | X | X | X |
| DEXA* |  | X |  |  |  |  |  |  |  |  |  |  |  |  | X |  |
| Quality of life questionnaire | X | X |  |  | X |  | X |  | X |  | X | X | X | X | X |  |
| Volume of blood drawn (ml) | 76 | 72 | 72 | 72 | 72 | 72 | 72 | 72 | 72 | 72 | 72 | 72 | 72 | 72 | 72 | 72 |

*) AMC only

**) hematology, including full blood count, leukocytes + differentiation, clinical chemistry, including liver enzymes

^†^) repeated until positive/complete/stable

^††^) suggested regular healthcare visits every three months

Visit schedule 15 month treatment group

| TRIAL PERIOD | Screening | Baseline |  |  |  |  |  |  |  | Interrupt |  |  |  |  |  |  |
| --- | --- | --- | --- | --- | --- | --- | --- | --- | --- | --- | --- | --- | --- | --- | --- | --- |
| WEEK | -4 TO –1 | 0 | 2 | 4 | 8 | 12 | 24 | 36 | 48 | 60 | 4 | 8 | 12 | 24 | 36 | Etc. ^††^ |
| Study duration (months) |  | 0 |  | 1 | 2 | 3 | 6 | 9 | 12 | 15 | 16 | 17 | 18 | 21 | 24 |  |
| Visit number | 1 | 2 | 3 | 4 | 5 | 6 | 7 | 8 | 9 | 10 | 1a | 2a | 3a | 4a | 5a |  |
| Eligibility criteria | X |  |  |  |  |  |  |  |  |  |  |  |  |  |  |  |
| Informed consent | X |  |  |  |  |  |  |  |  |  |  |  |  |  |  |  |
| Complete medical history | X |  |  |  |  |  |  |  |  |  |  |  |  |  |  |  |
| Prior antiretroviral therapy | X |  |  |  |  |  |  |  |  |  |  |  |  |  |  |  |
| Concomitant medications | X | X | X | X | X | X | X | X | X | X | X | X | X | X | X | X |
| Physical examination | X | X | X | X | X | X | X | X | X | X | X | X | X | X | X | X |
| Routine laboratory testing** | X | X | X | X | X | X | X | X | X | X | X | X | X | X | X | X |
| CD4^+^ / CD8^+^ lymphocytes | X | X | X | X | X | X | X | X | X | X | X | X | X | X | X | X |
| ELISA for HIV-1 antibody^†^ | X |  |  |  |  |  |  |  |  |  |  |  |  |  |  |  |
| Western blot^†^ | X |  |  |  |  |  |  |  |  |  |  |  |  |  |  |  |
| Genotypic resistance | X |  |  |  |  |  |  |  |  |  |  |  |  |  |  |  |
| Plasma HIV-1 RNA levels | X | X | X | X | X | X | X | X | X | X | X | X | X | X | X | X |
| Urine pregnancy test | X | X |  |  |  |  |  |  |  |  |  |  |  |  |  |  |
| Fasting plasma lipid spectrum* | X | X |  | X |  |  | X |  |  | X | X |  |  |  | X |  |
| Urine analysis* |  | X |  |  |  |  |  |  |  | X |  |  |  |  | X |  |
| Markers bone metabolism* |  | X |  |  |  |  |  |  |  | X |  |  |  |  | X |  |
| PBMC virology* | X |  |  |  |  |  | X |  | X |  |  |  |  | X |  |  |
| PBMC/plasma/serum storage* |  | X | X | X | X | X |  | X |  | X | X | X | X |  | X | X |
| DEXA* |  | X |  |  |  |  |  |  |  | X |  |  |  |  | X |  |
| Quality of life questionnaire | X | X |  |  | X |  | X | X | X | X |  | X | X | X | X |  |
| Volume of blood drawn (ml) | 76 | 72 | 72 | 72 | 72 | 72 | 72 | 72 | 72 | 72 | 72 | 72 | 72 | 72 | 72 | 72 |

*) AMC only

**) hematology, including full blood count, leukocytes + differentiation, clinical chemistry, including liver enzymes

^†^) repeated until positive/complete/stable

^††^) suggested regular healthcare visits every three months

Visit schedule no treatment group

| TRIAL PERIOD | Screening | Baseline |  |  |  |  |  |  |  |  |  |  |  |  |
| --- | --- | --- | --- | --- | --- | --- | --- | --- | --- | --- | --- | --- | --- | --- |
| WEEK | -4 TO –1 | 0 | 2 | 4 | 8 | 12 | 24 | 36 | 48 | 60 | 72 | 84 | 96 | Etc. ^††^ |
| Study duration (months) |  | 0 |  | 1 | 2 | 3 | 6 | 9 | 12 | 15 | 18 | 21 | 24 |  |
| Visit number | 1 | 2 | 3 | 4 | 5 | 6 | 7 | 8 | 9 | 10 | 11 | 12 | 13 |  |
| Eligibility criteria | X |  |  |  |  |  |  |  |  |  |  |  |  |  |
| Informed consent | X |  |  |  |  |  |  |  |  |  |  |  |  |  |
| Complete medical history | X |  |  |  |  |  |  |  |  |  |  |  |  |  |
| Prior antiretroviral therapy | X |  |  |  |  |  |  |  |  |  |  |  |  |  |
| Concomitant medications | X | X | X | X | X | X | X | X | X | X | X | X | X | X |
| Physical examination | X | X | X | X | X | X | X | X | X | X | X | X | X | X |
| Routine laboratory testing** | X | X | X | X | X | X | X | X | X | X | X | X | X | X |
| CD4^+^ / CD8^+^ lymphocytes | X | X | X | X | X | X | X | X | X | X | X | X | X | X |
| ELISA for HIV-1 antibody^†^ | X | X | X | X | X | X | X | X | X | X | X | X | X | X |
| Western blot^†^ | X | X |  | X | X | X | X |  |  |  |  |  |  |  |
| Genotypic resistance | X |  |  |  |  |  |  |  |  |  |  |  |  |  |
| Plasma HIV-1 RNA levels | X | X | X | X | X | X | X | X | X | X | X | X | X | X |
| Urine pregnancy test | X | X |  |  |  |  |  |  |  |  |  |  |  |  |
| Fasting plasma lipid spectrum* | X | X |  |  |  |  | X | X |  |  |  |  | X |  |
| Urine analysis* |  | X |  |  |  |  |  | X |  |  |  |  | X |  |
| Markers bone metabolism* |  | X |  |  |  |  |  | X |  |  |  |  | X |  |
| PBMC direct analysis* | X |  |  |  |  |  | X |  |  |  |  | X |  |  |
| PBMC/plasma/serum storage* |  | X | X | X | X | X |  | X | X | X | X |  | X | X |
| DEXA* |  | X |  |  |  |  |  |  |  |  |  |  | X |  |
| Quality of life questionnaire | X | X |  |  | X |  | X | X | X | X | X | X | X |  |
| Volume of blood drawn (ml) | 76 | 76 | 72 | 76 | 76 | 76 | 76 | 72 | 72 | 72 | 72 | 72 | 72 |  |

*) AMC only

**) hematology, including full blood count, leukocytes + differentiation, clinical chemistry, including liver enzymes

^†^) repeated until positive/complete/stable

^††^) suggested regular healthcare visits every three month

**References**

1. Rosenwirth,B. *et al.* Antiretroviral therapy during primary immunodeficiency virus infection can induce persistent suppression of virus load and protection from heterologous challenge in rhesus macaques. *J. Virol.* **74**, 1704-1711 (2000).

2. Rosenberg,E.S. *et al.* Immune control of HIV-1 after early treatment of acute infection. *Nature* **407**, 523-526 (2000).

3. Malhotra,U. *et al.* Effect of combination antiretroviral therapy on T-cell immunity in acute human immunodeficiency virus type 1 infection. *J. Infect. Dis.* **181**, 121-131 (2000).

4. Rosenberg,E.S. *et al.* Immune control of HIV-1 after early treatment of acute infection. *Nature* **407**, 523-526 (2000).

5. Fidler,S. *et al.* Virological and immunological effects of short-course antiretroviral therapy in primary HIV infection. *AIDS* **16**, 2049-2054 (2002).

6. Altfeld,M. *et al.* Cellular immune responses and viral diversity in individuals treated during acute and early HIV-1 infection. *J. Exp. Med.* **193**, 169-180 (2001).
